# Supplementary material for: The Systems Biology Research Tool: evolvable open-source software
Source: BMC Syst Biol. 2008 Jun 29;2:55. doi: 10.1186/1752-0509-2-55 (PMC2446383; doi:10.1186/1752-0509-2-55)
Supplement: Additional file 1 — SBRT Archive. An archive of the current version of the Systems Biology Research Tool. [file 1752-0509-2-55-S1.zip › sbrt-1.4.0/doc/developers_guide/api/sbrt/shell/io/SmoFile.html]

SmoFile


|  |  |  |
| --- | --- | --- |
| **Overview** \| **Package** \| **Class** \| **Use** \| **Tree** \| **Deprecated** \| **Index** \| **Help** | |  |
| **PREV CLASS**   **NEXT CLASS** | **FRAMES**    **NO FRAMES**     **All Classes** |  |
| SUMMARY: NESTED \| FIELD \| CONSTR \| METHOD | DETAIL: FIELD \| CONSTR \| METHOD |  |


---


## sbrt.shell.io Class SmoFile<K,V>

```
java.lang.Object
  sbrt.shell.io.SerializedOutputFile<java.util.Map<? extends K,? extends V>>
      sbrt.shell.io.SmoFile<K,V>
```

**Type Parameters:**: `K` - the key type.: `V` - the value type.

**All Implemented Interfaces:**: ResultsManager<java.util.Map<? extends K,? extends V>>, MapOutputFile<K,V>, OutputFile<java.util.Map<? extends K,? extends V>>, SbrtFile

---

``` public class SmoFile<K,V> extends SerializedOutputFile<java.util.Map<? extends K,? extends V>> implements MapOutputFile<K,V> ```

This class is used to write serialized maps to output files.
This class works in conjunction with `SerializedMapInputFile`.

**Author:**
:   This class was written and documented by
    Jeremiah Wright while in the Wagner lab.

---

**Constructor Summary**

`SmoFile(java.lang.String fileName,
FileFormat fileFormat,
java.util.Set<? extends K> allKeys)`
  
          Constructs a new serialized map output file from the
provided arguments.


| **Method Summary** | |
| --- | --- |
| `void` | `addData(java.util.Map<? extends K,? extends V> map)`             Writes the appropriate values of the provided map to this file. |
| `java.util.Set<K>` | `getFilterKeys()`             Returns the set of keys whose values will be written to this output file when a map is added. |

**Methods inherited from class sbrt.shell.io.SerializedOutputFile**

`addData, breakPoint, close, finalize, getCanonicalName, getFileFormat, getName, getOriginalName, isClosed, isEof, isValidFormat, writeObject`

**Methods inherited from class java.lang.Object**

`clone, equals, getClass, hashCode, notify, notifyAll, toString, wait, wait, wait`

**Methods inherited from interface sbrt.shell.io.SbrtFile**

`close, getCanonicalName, getOriginalName, isClosed`

**Methods inherited from interface sbrt.kernel.io.ResultsManager**

`addData, breakPoint, close, getName, isClosed`

**Constructor Detail**

### SmoFile

```
public SmoFile(java.lang.String fileName,
               FileFormat fileFormat,
               java.util.Set<? extends K> allKeys)
```

:   Constructs a new serialized map output file from the
    provided arguments.

    **Parameters:**: `fileName` - the name of the file to be created.: `fileFormat` - the format of the file to be created.: `allKeys` - the set of keys to be contained in this file. `null` is not allowed.


**Method Detail**

### getFilterKeys

```
public java.util.Set<K> getFilterKeys()
```

:   Returns the set of keys whose values will be
    written to this output file when a map is added.
    This set acts as a type of filter to control which
    values in a map are saved, and which are discarded.

    :   **Specified by:**: `getFilterKeys` in interface `MapOutputFile<K,V>`
    :   **Returns:**: the set of keys whose values will be written to this output file when a map is added.

---


### addData

```
public void addData(java.util.Map<? extends K,? extends V> map)
```

:   Writes the appropriate values of the provided map
    to this file. The appropriate values are those of the
    keys in the set returned by `getFilterKeys()`.

    :   **Specified by:**: `addData` in interface `MapOutputFile<K,V>`
    :   **Parameters:**: `map` - the map to be written to this file. **Throws:**: `java.lang.IllegalArgumentException` - if the provided map does not contain all the keys in the set returned by `getFilterKeys()`.: `java.lang.IllegalStateException` - if `close()` has already been called.


---


|  |  |  |
| --- | --- | --- |
| **Overview** \| **Package** \| **Class** \| **Use** \| **Tree** \| **Deprecated** \| **Index** \| **Help** | |  |
| **PREV CLASS**   **NEXT CLASS** | **FRAMES**    **NO FRAMES**     **All Classes** |  |
| SUMMARY: NESTED \| FIELD \| CONSTR \| METHOD | DETAIL: FIELD \| CONSTR \| METHOD |  |


---
